# Regulatory Approval, Reimbursement, and Clinical Use of Cyclin-Dependent Kinase 4/6 Inhibitors in Metastatic Breast Cancer in the Netherlands

**DOI:** 10.1001/jamanetworkopen.2022.56170

**Published:** 2023-02-16

**Authors:** Marianne Luyendijk, Hedwig Blommestein, Carin Uyl-de Groot, Sabine Siesling, Agnes Jager

**Affiliations:** 1 Department of Research and Development, Netherlands Comprehensive Cancer Organization, Utrecht, the Netherlands; 2 Erasmus School of Health Policy and Management, Erasmus University Rotterdam, Rotterdam, the Netherlands; 3 Department of Health Technology and Services Research, Technical Medical Centre, University of Twente, Enschede, the Netherlands; 4 Department of Medical Oncology, Erasmus MC Cancer Institute, Rotterdam, the Netherlands

## Abstract

**Importance:**

The number of new cancer medicines that are being approved by regulatory agents is increasing exponentially. Yet little is known about the pace at which these medicines reach eligible patients in daily clinical practice during different phases of the postapproval access pathway.

**Objective:**

To describe the entire postapproval access pathway of cyclin-dependent kinase 4/6 (CDK4/6) inhibitors in the Netherlands, from regulatory approval to reimbursement and to investigate the adoption of these medicines in clinical practice among patients with metastatic breast cancer.

**Design, Setting, and Participants:**

This cohort study reviewed approval and reimbursement decisions of the CDK4/6 inhibitors palbociclib, ribociclib, and abemaciclib and estimated the number of patients with metastatic breast cancer who were eligible for these medicines compared with the actual use in clinical practice. The study used nationwide claims data that were obtained from the Dutch Hospital Data. Claims and early access data for patients with hormone receptor–positive and *ERBB2* (formerly *HER2*)–negative metastatic breast cancer who were treated with CDK4/6 inhibitors from November 1, 2016, to December 31, 2021, were included.

**Main Outcomes and Measures:**

Description of the postapproval access pathway, monthly number of patients who were treated with CDK4/6 inhibitors in clinical practice, and estimated number of patients who were eligible for treatment. Aggregated claims data were used, and patient characteristics and outcomes data were not collected.

**Results:**

Three CDK4/6 inhibitors have received European Union–wide regulatory approval for the treatment of HR-positive and *ERBB2*-negative metastatic breast cancer since November 2016. In the Netherlands, the number of patients who have been treated with these medicines increased to approximately 1847 (based on 1 624 665 claims over the entire study period) from approval to the end of 2021. Reimbursement for these medicines was granted between 9 and 11 months after approval. While awaiting reimbursement decisions, 492 patients received palbociclib, the first approved medicine of this class, via an expanded access program. By the end of the study period, 1616 patients (87%) were treated with palbociclib, whereas 157 patients (7%) received ribociclib, and 74 patients (4%) received abemaciclib. The CKD4/6 inhibitor was combined with an aromatase inhibitor in 708 patients (38%) and with fulvestrant in 1139 patients (62%). The pattern of use over time appeared to be somewhat lower compared with the estimated number of eligible patients (1847 vs 1915 in December 2021), especially in the first 2.5 years after approval.

**Conclusions and Relevance:**

This study found that CDK4/6 inhibitors rapidly reached many eligible patients with metastatic breast cancer and were adopted gradually over time in the Netherlands. Adoption of innovative medicines may be further optimized, and better transparency of the availability of new medicines during different phases of the postapproval access pathway is needed.

## Key Points

**Question:**

How have cyclin-dependent kinase 4/6 (CDK4/6) inhibitors reached patients in need of these treatments in the Netherlands?

**Findings:**

In this retrospective cohort study, approximately 1847 patients have adopted the CDK4/6 inhibitors (palbociclib, ribociclib, and abemaciclib) by the end of the study in 2021. Although these medicines, particularly palbociclib, reached many Dutch patients who were eligible for this treatment, adoption rates were lower than expected in the first 2.5 years after approval.

**Meaning:**

Findings of the study suggest that, although CDK4/6 inhibitors rapidly reached many patients who were eligible for these treatments, the speed of adoption of innovative medicines may be further optimized and better transparency of the availability of these medicines is needed.

## Teaser

This cohort study examines the postapproval processes and timing for making palbociclib, ribociclib, and abemaciclib available to patients with metastatic breast cancer in a high-income country.

## Introduction

Over the past 2 decades, a range of new targeted medicines and immune therapies have been developed for different types of cancer.^1^ These pharmaceutical innovations have the potential to improve the outcomes of patients with cancer.^2^ However, this improvement can be realized only when all patients who may benefit have access to these new medicines at the right time.^3^ Within oncology, a gap has been observed between the rapid development of new medicines and their actual adoption in clinical practice.^4,5^

After innovative medicines are approved by regulatory authorities, there are several procedures that need to be completed before the drugs become available to patients. For instance, in most high-income countries, new expensive cancer medicines are subject to health technology assessment (HTA) procedures to guarantee that scarce resources are spent on treatments that offer value for money.^6^ Moreover, after reimbursement has been agreed on, treatment guidelines should be adapted, supply should be guaranteed, and clinicians need to prescribe the new medicines to eligible patients. These postapproval procedures take time and are often blamed for delayed or restricted patient access to innovative medicines.^7^

Even among the wealthiest countries in the world, achieving timely and sustained access to cancer drugs has been proven difficult.^5,8,9^ Monitoring access is thus desirable to identify possible delays in use and disparities in uptake and to assist with improvement. Previous studies on the access of patients with cancer to new medicines have covered different phases of the access pathway. For instance, some studies evaluated the degree to which new medicines were available, reimbursed, or subsidized in countries of varying economic status.^4,10,11,12^ Other studies evaluated the time from approval to first sales and described sales over time.^5,7^ These studies revealed delays in use and variations in access and uptake, but to our knowledge, no study has yet assessed the entire access pathway, from regulatory approval to reimbursement to adoption among eligible patients.

In this context, the cyclin-dependent kinase 4/6 (CDK4/6) inhibitors palbociclib, ribociclib, and abemaciclib, which are indicated for the treatment of patients with metastatic breast cancer, are interesting to study. These medicines were approved starting in 2016, and thus sufficient follow-up data are available for studying adoption rates over time. Moreover, these medicines offer renewed options for patients with hormone receptor (HR)–positive and *ERBB2* (formerly *HER2*)–negative metastatic breast cancer. With improvements of approximately 10 months in progression-free survival and up to 14 months in overall survival reported in randomized clinical trials (RCTs),^13,14^ these 3 CDK4/6 inhibitors are seen as important breakthroughs in the treatment of metastatic breast cancer.

In this cohort study, we evaluated the entire postapproval access pathway of CDK4/6 inhibitors from regulatory approval to reimbursement in the Netherlands, a high-income country in Europe, and investigated the adoption of these medicines in clinical practice. To identify the extent to which these medicines have reached eligible patients, we compared use with estimated use among eligible patients with metastatic breast cancer.

## Method

### Study Setting

In the Netherlands, different postapproval procedures need to be completed before newly approved, solid oncological medicines reach patients in need (eAppendix 1 and eFigure 1 in Supplement 1). First, a clinical committee formulates a recommendation regarding the use of the new medicine in clinical practice (clinical assessment). This advice can be interpreted as clinical guideline recommendations.^15^ Second, expensive medicines require an HTA. Depending on the results of these analyses, the Ministry of Health, Welfare and Sport in the Netherlands decides whether the medicines can be reimbursed.^16,17^ Third, if reimbursement is granted, the pharmacy should guarantee a sufficient supply and the government needs to ensure that declaration codes are available so that hospitals can bill insurance companies (logistic and administrative procedures). Ultimately, adoption in clinical practice should take place. New medicines may be available via alternative routes during the postapproval processes. This study was deemed exempt from review by Dutch legislation, which does not require informed consent from patients or approval by a medical ethics committee for this type of study, which did not contain directly identifiable patient data. We followed the Strengthening the Reporting of Observational Studies in Epidemiology (STROBE) reporting guideline.

### Study Approach and Data Sources

This retrospective cohort study comprised 3 parts: (1) a review of postapproval decisions for the CDK4/6 inhibitors, (2) data analyses of the number of patients who were treated in clinical practice, and (3) an estimation of the number of eligible patients. Aggregated claims data were used, but patient characteristics and outcomes data were not collected.

Two data sources, claims data and early access data, were consulted to identify the number of patients with HR-positive and *ERBB2*-negative metastatic breast cancer who were treated in clinical practice. First, the manufacturer of palbociclib provided data on the number of new patients (incident cases) who were treated each month during the wait for reimbursement. During this period of approximately 9 months, the manufacturer initiated an Expanded Access Program (EAP) for patients with HR-positive and *ERBB2*-negative metastatic breast cancer. The number of patients who received treatment (prevalent cases) per month were estimated (eAppendix 2 in Supplement 1). Second, we obtained monthly (May 2016-December 2021) claims data on the CDK4/6 inhibitors by indication from the nationwide Dutch Hospital Data, which covers almost all (approximately 98%) hospitals in the Netherlands. Indications in the data set were coded according to the European Medicines Agency (EMA) label, and the number of prescriptions were specified per dose. We included only the prescriptions for patients with HR-positive and *ERBB2*-negative metastatic breast cancer. These data were used to calculate the monthly number of prevalent cases treated with each of the 3 CDK4/6 inhibitors after reimbursement was granted. We divided the number of prescriptions per month with the expected number of pills per patient per 4 weeks (ie, 21 for palbociclib, 63 for ribociclib, and 56 for abemaciclib).

### Statistical Analysis

The monthly number of patients who were eligible based on the indications from the EMA label (Table 1) was estimated using a 2-step approach. First, we estimated the number of incident cases eligible for first- and second-line CDK4/6 inhibitor combined with endocrine therapy (ET) each month. We used incidence statistics of patients with breast cancer from the Netherlands Cancer Registry as a starting point. We then calculated the number of patients with HR-positive and *ERBB2*-negative metastatic breast cancer per treatment line by applying proportions that were obtained from the literature^18,19,20,21,22,23^ (ie, mainly a Dutch cohort study^21^; Table 2) (eAppendix 3 and eFigure 2 in Supplement 1). In the Netherlands, the recommendation is to prescribe CDK4/6 inhibitors in the second line of treatment.^24^ Thus, the only patients who were eligible for CDK4/6 inhibitors in the first line of treatment were those who developed metastases within 12 months after the end of adjuvant treatment with an aromatase inhibitor.^24^ In addition to the EMA label indications, we assumed that a proportion of patients may not be eligible to receive CDK4/6 inhibitors. These were patients who already underwent 2 lines of ET (mainly older adults); who died while receiving first-line ET; who had an aggressive course of the disease, and thus cannot undergo ET; and who did not want any anticancer treatment. We assumed that patients who did not meet the criteria for use of CDK4/6 inhibitors in the first or second line of treatment at the time of reimbursement would not receive them in a later line.

**Table 1.** EMA-Label Indications for Approved CDK4/6 Inhibitors in the Netherlands

CDK4/6 inhibitor	Therapeutic indication according to EMA product information
Palbociclib	Palbociclib is indicated for the treatment of patients with HR-positive and *ERBB2*-negative locally advanced or MBC in combination with an AI or in combination with fulvestrant in patients who received prior ET.
Ribociclib	Ribociclib in combination with letrozole is indicated as initial endocrine-based therapy for postmenopausal patients with HR-positive and *ERBB2*-negative advanced or MBC.
Extended-indication ribociclib	Ribociclib is indicated for patients with HR-positive and *ERBB2*-negative locally advanced or MBC in combination with an AI or fulvestrant as initial endocrine-based therapy or for patients who received prior ET. The population was extended to premenopausal patients and patients who received prior ET in combination with fulvestrant.
Abemaciclib	Abemaciclib is indicated for the treatment of patients with HR-positive and *ERBB2*-negative locally advanced or MBC in combination with an AI or fulvestrant as initial endocrine-based therapy or in patients who received prior ET.

Abbreviations: AI, aromatase inhibitor; CDK4/6, cyclin-dependent kinase 4/6; EMA, European Medicines Agency; ET, endocrine therapy; HR, hormone receptor; MBC, metastatic breast cancer.

**Table 2.** Estimated New Patient Eligibility for First- and Second-line CDK4/6 Inhibitors in the Netherlands

	No. of patients	Description	Source
**Diagnosis**			
BC	15 003	Total No. of new patients with invasive BC in 2017	Integraal Kankercentrum Nederland,^18^ 2021
MBC at primary diagnosis	750	5% Of patients had MBC	van der Meer et al,^19^ 2021
MBC after primary BC	2851	20% Of patients developed metastatic recurrence	Lobbezoo et al,^20^ 2015
HR-positive and *ERBB2*-negative MBC	2377	66% Of patients had HR-positive and *ERBB2*-negative disease	Lobbezoo et al,^21^ 2016
Total HR-positive and *ERBB2*-negative MBC	2377	Total No. of new patients with HR-positive and *ERBB2*-negative MBC	NA
**Eligibility for first-line ET with CDK4/6 inhibitors^a^**			
Developed metastases shortly after the end of or during adjuvant treatment with an AI	217	70% Of patients with HR-positive and *ERBB2*-negative MBC treated with first-line ET	Lobbezoo et al,^21^ 2016
		13% Of patients with metachronous disease developed metastases within 24 mo after the diagnosis of primary BC; these patients were assumed to have received an AI shortly before developing metastases and therefore were not eligible for ET combined with CDK4/6 inhibitor as second-line ET	Lobbezoo et al,^21^ 2016; assumption
Initially treated with chemotherapy	238	22% Of patients with HR-positive and *ERBB2*-negative MBC received chemotherapy as initial therapy	Lobbezoo et al,^21^ 2016
		56% Of patients were eligible for first-line ET with a CDK4/6 inhibitor^b^	Lobbezoo et al,^21^ 2016; Fietz et al,^22^ 2017; assumptions
		80% Of patients eligible for first-line ET after chemotherapy were assumed to receive ET combined with palbociclib as first-line ET	Assumption
Total eligible per y	455	Total No. of patients eligible for first-line ET combined with CDK4/6 inhibitor per year	NA
Total eligible per mo	38	Total No. of patients eligible for first-line ET combined with CDK4/6 inhibitor per month	NA
**Eligibility for second-line ET with CDK4/6 inhibitors^a^**			
Initially treated with ET	760	70% Of patients with HR-positive and *ERBB2*-negative MBC were treated with first-line ET	Lobbezoo et al,^21^ 2016
		87% Of patients were not eligible for first-line CKD4/6 inhibitors^c^	Lobbezoo et al,^21^ 2016
		58% Of patients were eligible for another line of ET^d^	Rugo et al,^23^ 2019
		90% Of patients were willing or able to use CDK4/6 inhibitors	Assumption
Initially treated with ET followed by a line of chemotherapy	121	70% Of patients with HR-positive and *ERBB2*-negative MBC were treated with first-line ET; 87% of patients were not eligible for first-line CKD4/6 inhibitors^c^	Lobbezoo et al,^21^ 2016
		34% Of patients received chemotherapy after a line of ET	Assumptions
		35% Of patients were assumed to be eligible for second-line ET^e^	Fietz et al,^22^ 2017; assumptions
		70% Of patients were eligible for second-line ET combined with CDK4/6 inhibitors	Assumption
Initially treated with chemotherapy followed by ET	15	22% Of patients with HR-positive and *ERBB2*-negative MBC received chemotherapy as initial therapy	Lobbezoo et al,^21^ 2016; Fietz et al,^22^ 2017
		56% Of patients were eligible for first line ET with CDK4/6^b^	Lobbezoo et al,^21^ 2016; Fietz et al,^22^ 2017; assumptions
		20% Of patients were assumed to not receive a first line of CDK4/6 inhibitors combined with ET after chemotherapy^f^	Assumption
		35% Of patients were assumed to be eligible for second-line ET^e^	Rugo et al,^23^ 2019
		70% Of patients were eligible for second-line ET combined with CDK4/6 inhibitors	Assumption
Total eligible per y	895	Total No. of patients eligible for first-line ET combined with CDK4/6 inhibitor per year	
Total eligible per mo	75	Total No. of patients eligible for first-line ET combined with CDK4/6 inhibitor per month	

Abbreviations: AI, aromatase inhibitor; BC, breast cancer; CDK4/6, cyclin-dependent kinase 4/6; ET, endocrine therapy; HR, hormone receptor; MBC, metastatic breast cancer; NA, not applicable.

^a^ We distinguished lines of ET from lines of chemotherapy (eg, patients who had 1 line of chemotherapy are still eligible for first-line CDK4/6 inhibitor combined with ET because it is their first line of ET).

^b^ This percentage was calculated based on the assumption that 26% would receive second-line chemotherapy^21^ and 18% would not receive any therapy.^22^

^c^ Calculated as 100% minus 13% (of patients with metachronous disease who were assumed to have received an AI shortly before developing metastases and therefore were not eligible for ET combined with CDK4/6 inhibitor as second line).

^d^ This percentage was based on the PALOMA-2 (A Study of Palbociclib + Letrozole vs Letrozole for 1st Line Treatment of Postmenopausal Women With ER+/HER2- Advanced Breast Cancer) trial of palbociclib combined with letrozole in which 58% of patients treated with placebo (ET only) received ET as subsequent treatment.

^e^ Calculated as 100% minus 32% of patients who received another line of chemotherapy minus 33% of patients who died.

^f^ Calculated as 100% minus 80% of patients who were eligible for first-line ET after chemotherapy and assumed to receive ET combined with palbociclib as first line.

Second, we estimated the number of prevalent cases per month by summing up the incident cases of each new month and subtracting the number of patients who experienced progression. Information regarding the probability of progression was obtained from Kaplan-Meier curves from RCTs that evaluated the efficacy of palbociclib.^25,26^ We assumed that the efficacy of the other CDK4/6 inhibitors (ribociclib and abemaciclib) was similar. Because the reported follow-up in the RCTs was too short, we extrapolated the survival curves beyond the observed survival time using the approach of Hoyle and Henley^27,28^ (eAppendix 3 and eFigures 3-6 in Supplement 1). Finally, we calculated adoption rates by dividing the number of patients who were treated with the estimated number of patients who were eligible.

To represent the implications of uncertainty for the number of patients, we calculated upper and lower bounds of the base case using more conservative or optimistic numbers of incident cases. Data were analyzed in Stata, version 17 (StataCorp LLC); Microsoft Excel, version 2016 (Microsoft Corp); and R, version 4.2.2 (R Foundation for Statistical Computing).

## Results

**Figure 1.**
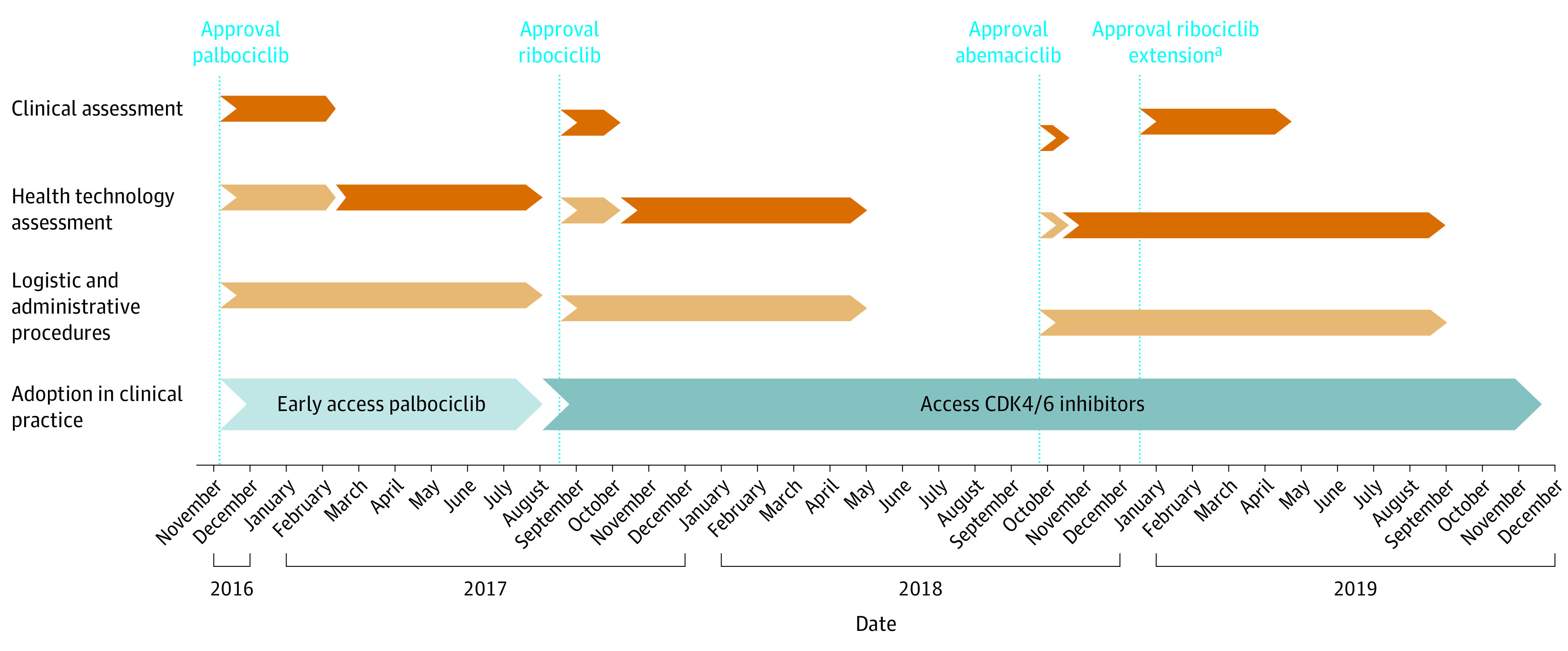
Postapproval Phases Needed for Access to Cyclin-Dependent Kinase 4/6 (CDK4/6) Inhibitors Postapproval phases were possible patient access delay (orange); possible delay overlapping with another phase (light orange); early access possibly not available to all patients (light blue); and access to reimbursed care (blue). Palbociclib combined with aromatase inhibitor in first-line treatment or with fulvestrant in second-line treatment. Ribociclib combined with letrozole in first-line treatment. Abemaciclib combined with aromatase inhibitor or with fulvestrant in first- or second-line treatment. Extended-indication ribociclib combined with fulvestrant in first- or second-line treatment. ^a^There was no additional health technology assessment because the extension was already considered in the initial decision.

Three CDK4/6 inhibitors have received European Union–wide regulatory approval for the treatment of HR-positive and *ERBB2*-negative metastatic breast cancer since November 2016. In the Netherlands, the number of patients who have been treated with these medicines increased to approximately 1847 (based on 1 624 665 claims over the entire study period) from approval to the end of 2021. Palbociclib received EMA approval in November 2016; ribociclib received initial EMA approval in August 2017, and its extended indication was approved in December 2018; and abemaciclib received EMA approval in September 2018. After regulatory approval, all 3 medicines were recommended for clinical use, but reimbursement decisions were postponed. After price negotiations, reimbursements were granted for palbociclib in August 2017, ribociclib in May 2018, and abemaciclib in September 2019. The clinical assessment phase and HTA phase lasted 3 and 9 months, respectively, for palbociclib; 1 month (first use) and 8 months for ribociclib; and 3 months and 11 months for abemaciclib (Figure 1).

### Early Access Phase

While awaiting clinical practice recommendations and reimbursement, 492 patients with HR-positive and *ERBB2*-negative metastatic breast cancer were treated with palbociclib via an alternative access route (ie, EAP). The number of new patients (incident cases) who participated in this EAP increased the first 3 months (from 24 to 60 patients), remained stable in the 5 months thereafter (from 52 to 65 patients), and decreased in the last month (from 65 to 40 patients) of the early access phase.

### Access Phase

**Figure 2.**
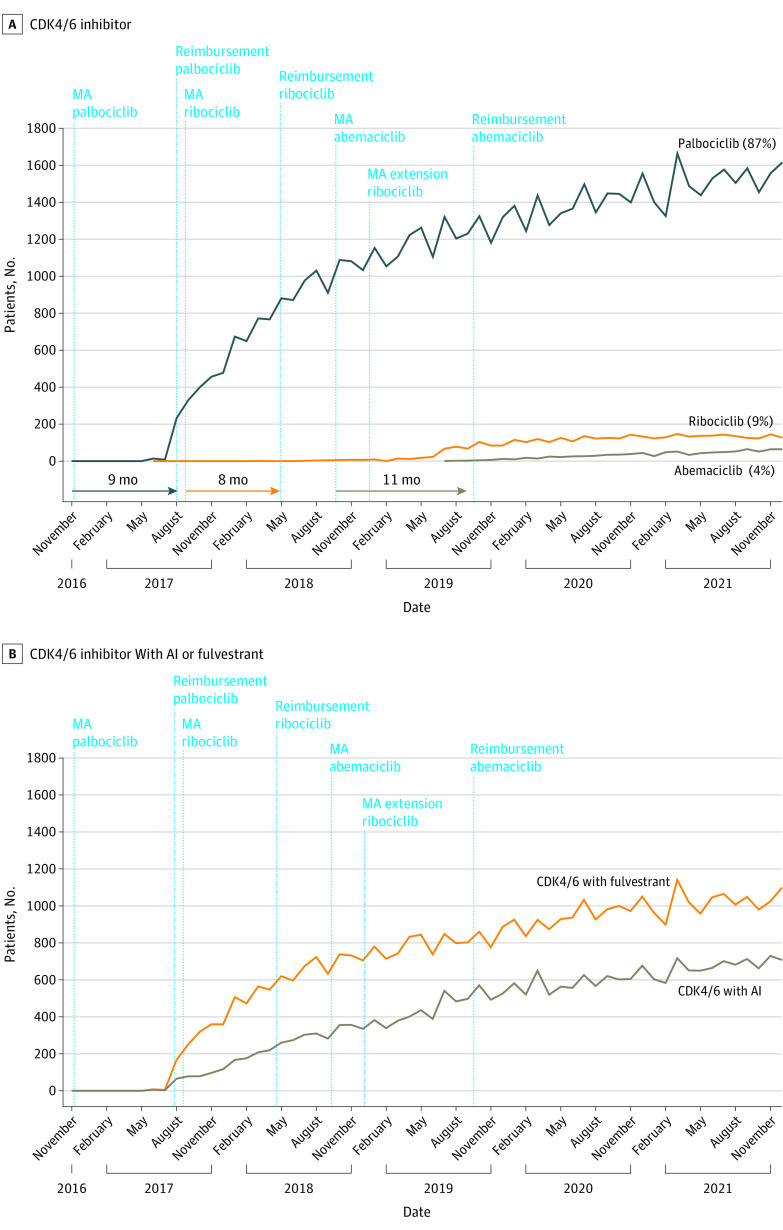
Observed Number of Patients Treated With 3 Cyclin-Dependent Kinase 4/6 (CDK4/6) Inhibitors and With a CDK4/6 Inhibitor Combined With Aromatase Inhibitor (AI) or Fulvestrant Over Time Palbociclib combined with AI in first-line treatment or with fulvestrant in second-line treatment. Ribociclib combined with letrozole in first-line treatment. Abemaciclib combined with AI or with fulvestrant in first- or second-line treatment. Extended-indication ribociclib combined with fulvestrant in first- or second-line treatment; there was no additional health technology assessment because the extension was already considered in the initial decision. MA indicates marketing authorization.

In the access phase, the adoption of the first approved and reimbursed CDK4/6 inhibitor, palbociclib, in clinical practice increased over time from 0 patients being treated to approximately 1847 patients over a period of more than 50 months. Use of palbociclib increased steeply after reimbursement was acquired. By the end of the study period, 1616 patients (87%) were treated with palbociclib. Only a small number of patients were treated with the other medicines of this class, ribociclib (n = 157 [7%]) and abemaciclib (n = 74 [4%]), by the end of the study period. A majority of patients (1139 [62%]) received CDK4/6 inhibitors in combination with fulvestrant, and 708 patients (38%) received it with an aromatase inhibitor (Figure 2).

### Entire Postapproval Phase

**Figure 3.**
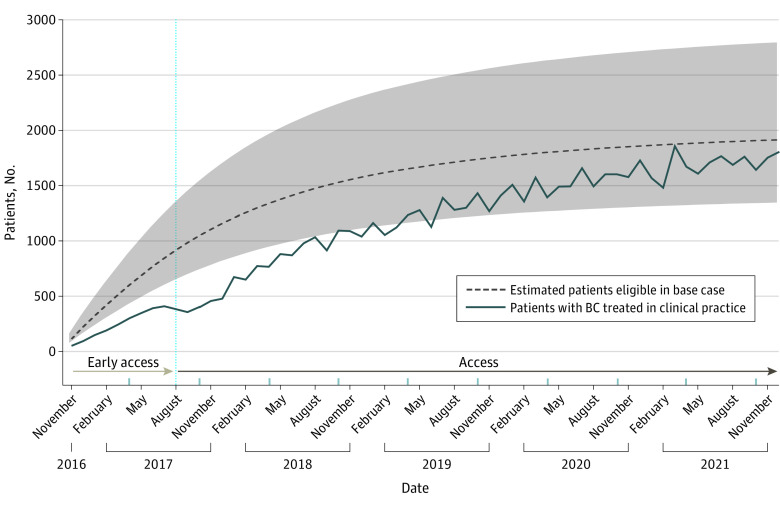
Observed Total Number of Patients Treated and Estimated Number of Prevalent Patients Eligible The shaded area indicates the upper and lower bounds of uncertainty for the estimated patients eligible in the base case. Early access is access to cyclin-dependent kinase 4/6 inhibitors before approval. BC indicates breast cancer.

The estimated number of patients eligible (prevalent cases) who were treated with CDK4/6 inhibitors increased during the study period, in the base case analysis, from approximately 110 patients in the month that regulatory approval was granted (November 2016) to approximately 1915 patients in the final month of the study (December 2021) (Figure 3). The pattern of use over time appeared to be somewhat lower compared with the estimated number of eligible patients, especially in the first 2.5 years after approval. The estimated adoption (ie, number of patients who were treated divided by estimated number of patients who were eligible) of the CDK4/6 inhibitors increased from approximately 383 of 918 (42%) directly after reimbursement was granted (August 2017) to approximately 1847 of 1915 patients (96%) at the end of the study period. We observed a steep increase in the number of patients who received treatment when we considered only the numbers calculated based on the claims data (8 patients before August 2017 to 880 in May 2018) (Figure 2). In contrast, a notable decline in the use (from approximately 410 patients in July 2017 to 357 in September 2017) and adoption (from approximately 42% to 36%) of CDK4/6 inhibitors was observed when we took into account that patients were already treated as part of the EAP in the early access phase (Figure 3).

### Sensitivity Analyses

In the base case, of the 198 incident cases, we estimated that 38 (19%) were eligible for first-line and 75 (38%) for second-line CDK4/6 inhibitors each month. Additionally, 85 incident cases (43%) were estimated to be ineligible (Table 2). We created 2 scenarios. First, we assumed that 119 patients (60%) would be ineligible for CDK4/6 inhibitors. Second, we assumed that 34 patients (17%) would be ineligible. The outcomes of these alternative assumptions are shown in Figure 3.

## Discussion

This cohort study showed that CDK4/6 inhibitors were gradually adopted in clinical practice in the Netherlands despite reimbursement decisions lagging 9 to 11 months behind the EMA approvals. The medicines reached many Dutch patients with breast cancer within a short period, but the number of patients who were treated in daily practice appeared to be somewhat lower than the estimated number of patients who were eligible in the first 2.5 years after regulatory approval.

While previous studies have reported delays of 9 months, on average, between the approval of cancer drugs and their first use in the Netherlands (2017-2020), the present study found that many patients received palbociclib immediately after regulatory approval was granted.^5,29^ Almost 500 patients participated in an EAP, which allowed them to benefit from this medicine even though it was not being reimbursed yet. This observation emphasizes 2 important points. First, it suggests that HTA procedures do not necessarily delay patient access to new medicines. This finding builds on earlier work that reported that different early access routes, either privately or publicly funded, offered options to patients in high-income countries.^30,31^ Second, it suggests that frequently used quality indicators of medicine accessibility (eg, time to reimbursement) should be interpreted with caution. These indicators are used not only to rank European countries according to their medicine accessibility but also to urge policy makers to improve accessibility.^5,29,32^ Based on the study findings, in-depth studies that evaluate the entire postapproval access pathway are needed to identify and elucidate the disparities in access to innovative medicines.

Despite the rapid use of palbociclib, there are some caveats worth mentioning with regard to its early availability via an EAP. First, there is little transparency about the availability and use of EAP programs in most countries, including the Netherlands.^33^ Because manufacturers are not allowed to advertise EAPs, not all clinicians may be aware of these programs. Lack of awareness can be a factor in inequalities in access between patients being treated by different physicians. Second, EAPs are not funded by the government in the Netherlands. Their implementation thus relies on the efforts and goodwill of pharmaceutical companies. While some companies may see benefits in EAPs (for instance, to speed up future sales), others may be unwilling or unable to allocate resources for implementing such programs.^34,35,36,37^ Because HTA procedures take time to complete, this delay likely plays a role in inequalities in access between different groups of patients. Countries surrounding the Netherlands have used different approaches to funding early access. For instance, in Germany, all newly EMA-approved medicines are reimbursed by default at the price set by the company, which ensures immediate access for patients. Pricing negotiations take place after the medicines have been launched.^37^ In France, the government funds different EAPs to allow patients to use medicines before reimbursement and marketing authorization.^30^ It remains unclear which approaches best deliver timely, sustainable, and affordable access to innovative medicines for all patients in need.

We evaluated the use of 3 medicines of the same class that were approved for combination with either an aromatase inhibitor or fulvestrant. In this study, we observed that palbociclib, the first approved medicine of the 3 CDK4/6 inhibitors, remained the predominant medicine that was prescribed. This preference for palbociclib was also seen in other high-income countries, although there is no clear evidence that palbociclib is superior to ribociclib and abemaciclib.^38,39,40,41,42^ Possibly the pattern of use can be explained by physician preferences, the marketing strategies of the manufacturers, hospital policies, or differences in price rebates. We also observed that the CDK4/6 inhibitors were mainly combined with fulvestrant, which likely reflects the recommendation in the Netherlands to primarily prescribe CDK4/6 inhibitors in the second line of treatment.^24^ The Dutch recommendation is contrary to that of other high-income countries (eg, the US), where they advise to prescribe CDK4/6 inhibitors in the first line of treatment.^43^ First-line treatment with these drugs has been associated with a longer progression-free survival but also with more toxic effects and higher costs due to longer treatment durations.^44^ Undoubtedly, the recommendation to use the CDK4/6 inhibitors mainly in the second line of treatment is interesting from a budget-impact point of view (ie, lower total costs), which may help to ensure sustainable health care financing and prevent access problems in the long run. It remains to be seen what strategy is the most optimal from the effectiveness and cost-effectiveness perspectives. This topic is currently being investigated in the Dutch SONIA (Endocrine Therapy Plus CDK4/6 in First or Second Line for Hormone Receptor Positive Advanced Breast Cancer) trial.^44^

It is well known that the adoption of innovations in clinical practice takes time.^45,46^ Therefore, it may be unsurprising that we observed a gap between the number of patients who were treated and the estimated number of patients who were eligible in the first 2.5 years after reimbursement was permitted. The disparities found in this study may partly reflect the uncertainty surrounding the estimated patient numbers, which relied on modeling rather than clinical practice data. However, we consider it plausible that the adoption of the 3 CDK4/6 inhibitors started more slowly than expected. Specifically, we observed a minor decline in the increasing use pattern from the moment that the EAP ceased and reimbursement was approved (August 2017). This decrease suggests that not all physicians responded directly to the changed reimbursement status of palbociclib. Raising awareness of this issue among treating physicians may further optimize the implementation of new medicines in the future.

### Limitations

This study has some limitations. First, the number of patients who were eligible for the 3 medicines relied on decision analytic modeling, and the scenario analyses we performed showed that the results had substantial amount of uncertainty. Thus, the estimated number of eligible patients and adoption rates should be interpreted with caution. To fully understand access, detailed information is needed regarding patients with metastatic breast cancer, their treatments, and the decision-making process in daily practice. Second, we used aggregated claims data to evaluate the use of the CDK4/6 inhibitors, which means that we did not have data on patient characteristics or outcomes. As such, we could not assess whether patients who received the CDK/6 inhibitors immediately after approval and reimbursement differed from those who accessed the medicines later. Third, we studied the adoption of only 1 medicine class for the treatment of breast cancers in a single country. Future studies need to compare the adoption of medicines across different countries with different HTA policies. Fourth, the COVID-19 crisis was ongoing during the study period (2020-2021). Nevertheless, we did not expect a large association between the crisis and study results because treatment recommendations for patients with metastatic disease during the pandemic were similar to those from the years before.^47^

## Conclusions

This cohort study found that the CDK4/6 inhibitors palbociclib, ribociclib, and abemaciclib were rapidly accessible to a group of patients with HR-positive and *ERBB2*-negative metastatic breast cancer and were adopted gradually over time in the Netherlands, a high-income country. Although many patients received the medicines during the period when reimbursement was pending, there appeared to be barriers to adoption other than reimbursement. To improve accessibility of innovative medicines, we call for better transparency of their availability during the different phases of the postapproval access pathway and for more in-depth cross-country studies on the adoption of medicines, which may further optimize the process.

## Notes

Supplement 1. **eAppendix 1.** Detailed Description of the Procedural Pathway of New Oncology Medicines in the Netherlands **eFigure 1.** Overview of the Different Post Approval Phases Needed to Acquire Access to New Expensive Anti-Cancer Medicines in the Netherlands **eAppendix 2.** Details About the Calculations Made to Identify the Number of Patients Treated Each Month 5 in the Expanded Access Program **eAppendix 3.** Details About the Estimates of Patients Eligible for Treatment per Month **eFigure 2.** Estimated Number of New (Incident) Patients Eligible for First and Second Line Treatment With CDK4/6 Inhibitors, Each Month **eFigure 3.** Original Kaplan-Meier Curves of the PALOMA-2 **eFigure 4.** Simulated Kaplan-Meier Curves of the PALOMA-2 and the Extrapolated Curves Based on Different Distributions **eFigure 5.** Original Kaplan-Meier Curves of the PALOMA-3 **eFigure 6.** Simulated Kaplan-Meier Curves of the PALOMA-3 and the Extrapolated Curves Based on Different Distributions
eReferences

Supplement 2.
Data Sharing Statement
