# Supplementary material for: Regulatory Approval, Reimbursement, and Clinical Use of Cyclin-Dependent Kinase 4/6 Inhibitors in Metastatic Breast Cancer in the Netherlands
Source: JAMA Netw Open. 2023 Feb 16;6(2):e2256170. doi: 10.1001/jamanetworkopen.2022.56170 (PMC9936344; doi:10.1001/jamanetworkopen.2022.56170)
Supplement: Supplement 1. — eAppendix 1. Detailed Description of the Procedural Pathway of New Oncology Medicines in the Netherlands eFigure 1. Overview of the Different Post Approval Phases Needed to Acquire Access to New Expensive Anti-Cancer Medicines in the Netherlands eAppendix 2. Details About the Calculations Made to Identify the Number of Patients Treated Each Month 5 in the Expanded Access Program eAppendix 3. Details About the Estimates of Patients Eligible for Treatment per Month eFigure 2. Estimated Number of New (Incident) Patients Eligible for First and Second Line Treatment With CDK4/6 Inhibitors, Each Month eFigure 3. Original Kaplan-Meier Curves of the PALOMA-2 eFigure 4. Simulated Kaplan-Meier Curves of the PALOMA-2 and the Extrapolated Curves Based on Different Distributions eFigure 5. Original Kaplan-Meier Curves of the PALOMA-3 eFigure 6. Simulated Kaplan-Meier Curves of the PALOMA-3 and the Extrapolated Curves Based on Different Distributions eReferences [file jamanetwopen-e2256170-s001.pdf]

Supplemental Online Content

Luyendijk M, Blommestein H, Uyl-de Groot C, Siesling S, Jager A. Regulatory approval, reimbursement, and clinical use of cyclin-dependent kinase 4/6 inhibitors in metastatic breast cancer in the Netherlands. *JAMA Netw Open*. 2023;6(2):e2256170. doi:10.1001/jamanetworkopen.2022.56170

eAppendix 1. Detailed Description of the Procedural Pathway of New Oncology Medicines in the Netherlands

eFigure 1. Overview of the Different Post Approval Phases Needed to Acquire Access to New Expensive Anti-Cancer Medicines in the Netherlands

eAppendix 2. Details About the Calculations Made to Identify the Number of Patients Treated Each Month 5 in the Expanded Access Program

eAppendix 3. Details About the Estimates of Patients Eligible for Treatment per

Month

eFigure 2. Estimated Number of New (Incident) Patients Eligible for First and Second Line Treatment With CDK4/6 Inhibitors, Each Month

eFigure 3. Original Kaplan-Meier Curves of the PALOMA-2

eFigure 4. Simulated Kaplan-Meier Curves of the PALOMA-2 and the Extrapolated Curves Based on Different Distributions

eFigure 5. Original Kaplan-Meier Curves of the PALOMA-3

eFigure 6. Simulated Kaplan-Meier Curves of the PALOMA-3 and the Extrapolated Curves Based on Different Distributions

eReferences

This supplementary material has been provided by the authors to give readers additional information about their work.

© 2023 Luyendijk M et al. *JAMA Network Open.*

eAppendix 1. Detailed Description of the Procedural Pathway of New Oncology Medicines in the Netherlands

The first phase of the access pathway is the regulatory approval phase. New medicines or new uses of existing once must receive regulatory approval by international authorities (the European Medicine Agency (EMA) and the European Commission (EC) to be used in the European Union (EU).

The second phase is specifically for new anti-cancer medicines for solid tumors and involves a thorough assessment of the clinical value of the drug by a committee . This committee formulates a positive or negative advice which can be interpreted as clinical guideline recommendations.[1]

The third phase involves the health technology assessment (HTA) phase. In the Netherlands, new medicines require a full HTA, including a analyses of the relative effectiveness and cost-effectiveness of the new drug compared to existing alternatives, when they are considered expensive. Specifically, 1) if the expected budget impact ≥40 million euro's per year or; 2) if the expected budget ≥10 million and the costs of treating one patient per year ≥50 thousand euros.[2,3] During the HTA period, new high price and or high budget-impact medicines are not included in the basic benefit package. The HTA evaluations may overlap with the clinical assessment (phase 2) but effectiveness evaluations are done in both phases. [3,4]

Finally, certain administrative procedures are required to ensure that medicines can be prescribed by treating oncologists and billed to insurance companies. In the Netherlands, medicines that costs over €1000 per patient per year can be billed by hospitals separately from other care via a specific add-on declaration code which must be requested at the Dutch Health Authority. [5]

Besides the availability of medicines via the conventional route (reimbursed care), access to new medicines is sometimes also possible via alternative routes. Prior to approval, patients may enroll in clinical trials or access unauthorized medicines via a compassionate use programs (CUPs) or on an named patient basis (NP). Sometimes, such programs are extended when new medicines are approved but the reimbursement decision is pending.

© 2023 Luyendijk M et al. *JAMA Network Open.*

eFigure 1. Overview of the Different Post Approval Phases Needed to Acquire Access to New Expensive Anti-Cancer Medicines in the Netherlands

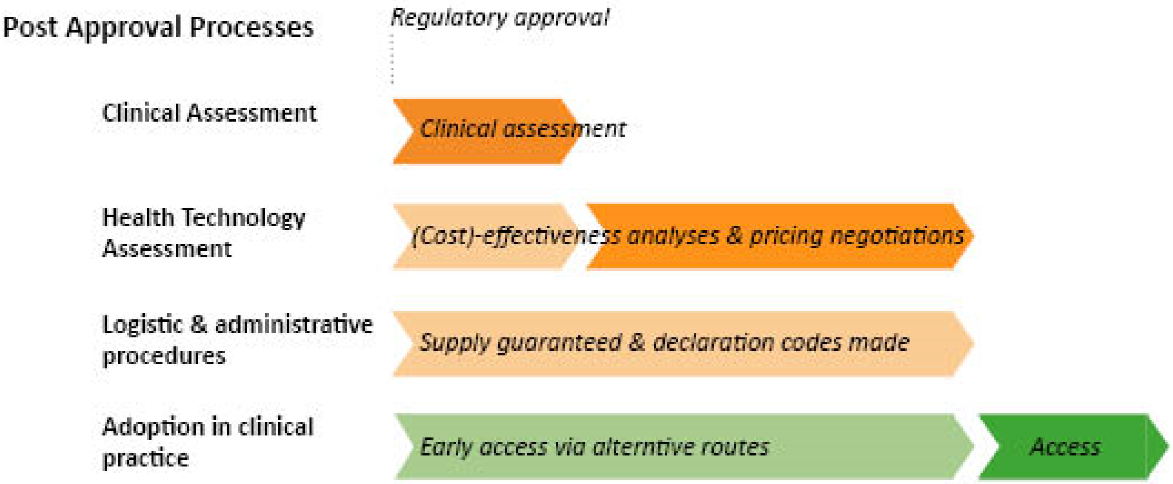

**Expensive is defined as medicines with an estimated; i) total financial impact >10 million euro per year for a single new indication; ii) total financial impact >40 million euro per year for one or multiple new indications; iii) when the costs of treatment exceed 50 thousand euro per patient per year.[16] MA: marketing authorization. Orange arrow: represents a period of possible delay in patient access; Light orange: represents a period of possible delay but overlap with another approval phase; Light green; represents early patient access that may not be available for all patients in need; Green: represents patient access to reimbursed care.*

© 2023 Luyendijk M et al. *JAMA Network Open.*

eAppendix 2. Details About the Calculations Made to Identify the Number of Patients Treated Each Month in the Expanded Access Program

The manufacturer provided us the number of new patients included in the expanded access programme (EAP) each month (‘incident cases’). Because we were interested in the total number of patient treated each month (‘prevalent cases’), we summed up the incident cases of each new month and subtracted patients that experienced progression. Since we did not receive information about the number of patients that experienced progression, we estimated these using probabilities obtained from published randomized controlled trials (RCTs). The specific approach used is to calculate the probabilities of progression is described in eAppendix 3.

© 2023 Luyendijk M et al. *JAMA Network Open.*

eAppendix 3. Details About the Estimates of Patients Eligible for Treatment per Month

One objective of our study was to compare patients treated in clinical practice with a CDK4/6 to the patients theoretically eligible for these drugs given the approved indication. The latter numbers had to be estimated because they are not known. We used different sources of evidence including literature and expert opinion. We estimated a 'base case' and upper and lower bound to present uncertainty. The base case represents the most likely situation, the upper and lower bound represent the range in which we believe the true values lay.

A two-step approach was used to estimate the number of patients theoretically eligible for a drug per month. First, we estimated the number of new patients (incident cases) eligible for a specific drug per month, both for the treatment in first and second line setting. Second, for each new month after the month of approval we added the new patients (estimated in step 1) and subtracted the number of patients that experienced progression (prevalent cases). Information on progression or death was obtained from the published Kaplan-Meier curves from the pivotal clinical trials of palbociclib (PALOMA 2 & 3).[6,7] The survival curves were extrapolated beyond the observed survival time because the we needed estimates of progression for the entire period of our study (form November 2016 to December 2021). For this purpose the approach of Hoyle & Henley was used.[8,9] We assumed that patients treated with palbociclib as first line treatment, would experience progression according to the PALOMA 2 (i.e. palbociclib + aromatase inhibitor (AI) is approved for the first line setting) and patients treated with palbociclib as second line treatment would experience progression according to the PALOMA 3 (i.e. Palbociclib + fulvestrant is approved for the use in patients who had prior ET).[6,7]

<u>CDK4/6 inhibitors for advanced/metastatic breast cancer – incident cases</u>

CDK4/6 inhibitors are approved for the treatment of hormone receptor positive and human epidermal growth factor receptor negative (HR+/HER2-) advanced or metastatic breast cancer combined with an AI or fulvestrant, either as initial endocrine therapy (ET) or in women who have received prior ET (for details see Table 1 original article). The professional association decided that CDK4/6 inhibitors should be applied as later line ET as much as possible.[10]

We first estimated the number of new patients with HR+HER2- metastatic breast cancer (mBC). Thereafter we estimated the number of new patients eligible for CDK4/6 as first line and as later line ET

© 2023 Luyendijk M et al. *JAMA Network Open.*

for their metastatic disease. eFigure 2 provides a schematic overview of our calculations and a detailed explanation is given below the figure.

© 2023 Luyendijk M et al. *JAMA Network Open.*

eFigure 2. Estimated Number of New (Incident) Patients Eligible for First and Second Line Treatment With CDK4/6 Inhibitors, Each Month

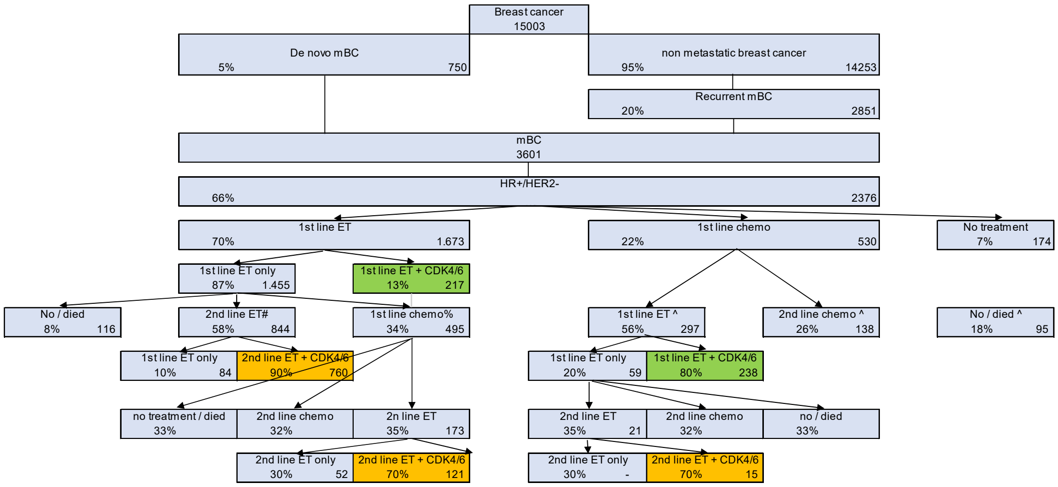

	Per year	Per month
Total 1st line CDK4/6	455	38
Total 2nd line CDK4/6	895	75

#Rugo et al.[11] ^Lobbezoo et al. [12]

Number of new patients with HR+/HER2- breast cancer

The Netherlands Cancer registry reports the number of new patients with invasive breast cancer per year. In 2017, 15,003 new patients were diagnoses with invasive breast cancer. Approximately 5% of these patients had metastatic breast cancer at initial presentation (de novo).[13] In addition, of the patients with non-metastatic breast cancer (N=14,253), approximately 20% experience a recurrence of their disease at which they develop distant metastases (recurrent). Altogether, these are approximately 3601 new patients with breast cancer and distant metastases each year. Based on a Dutch cohort study we know that approximately 66% of mBC patients has HR+/HER2- disease.[12] This study includes all patients with metastatic breast cancer in the south east of Netherlands and we assume that this cohort is representative for the Dutch general population. This brings us to 2377 patients with HR+/HER2- mBC.

Note: The numbers and proportions used for the above estimates are in accordance with estimates made by the ZIN (HTA agency of the Netherlands) to estimates the budget impact of palbociclib.[14]

Number of new patients eligible for first line therapy with CDK4/6

© 2023 Luyendijk M et al. *JAMA Network Open.*

Prior to the advent of CDK4/6 inhibitors, patients with HR+/HER2- mBC could be treated with either ET or chemotherapy. In addition, a minority of patients would not receive treatment at all. Patients that would be treated with ET previously are those eligible for ET + CDK4/6. According to a Dutch cohort study off Lobbezoo et al. 70%, 22% and 7.3% of patients were treated with ET (first line ET), chemotherapy (first line chemotherapy) and no therapy, respectively.[12] We therefore estimated that 70% of patients would be eligible for ET+CDK4/6 inhibitors but assumed that only 13% of those patients would actually be treated with CDK4/6+ET in first line and the remaining 57% would be treated with ET monotherapy (n=1455). This assumption is made because in the Netherlands it is decided that CDK4/6 inhibitors are only given in the first line of ET to patients who cannot get CDK4/6 in second or later line of ET. Generally, these are the patients who have received ET with Ais for their primary cancer and experienced a recurrence of their breast cancer within approximately two years. In the study of Lobbezoo et al. these were 13% of patients treated with ET. As such, we assume that a total of (13% of 70% =) 217 patients will be treated with first line CDK4/6 + ET.[12]

Another group of patients that is eligible for first line treatment with ET + CDK4/6 are the patients initially treated chemotherapy for their metastatic disease. We estimated that approximately 56% of the 22% patients treated with chemotherapy (see previous paragraph) as first line would be eligible for first line ET. In addition, we assumed that of these 297 patients, 80% would receive ET + CDK4/6 (n= 238) as first line ET therapy. The 56% is calculated based on the assumption that 26% would receive 2nd line chemotherapy and 18% would not receive any therapy. These assumptions are based on the Dutch study of Lobbezoo et al and a German cohort study of Fietz.[15] The study of Fietz et al report that approximately 18% of patients with HR+/HER2- mBC dies or is lost to follow-up before (figure 1A) and thus do not receive second line treatment.[15] 26% of patients in the study of Lobbezoo are expected to be receiving second line chemotherapy.[12] In addition, based on expert opinion we assumed that approximately 80% of the 56% of patients who are eligible for first line ET after a line of chemo, would receive ET+palbociclib as first line ET, which brings us to another 238 patients treated with first line ET + CDK4/6.

In total 217 + 238 = 455 new patients are estimated to be eligible and treated with first line ET+ CDK4/6 inhibitors each year implying 38 patients per month.

Note: The numbers and proportions of patients deviate from those estimated by ZIN, reason for this is that the ZIN assumed that all patients would receive ET + CDK4/6 in the first line except for those treated with chemotherapy as first line therapy. According to the consulted expert this is not realistic as

© 2023 Luyendijk M et al. *JAMA Network Open.*

the Dutch association of medical oncologist agreed to use CDK4/6 inhibitors as much as possible in the second line.[10] Moreover, the ZIN did not take into account that in HR+/HER2 negative treatment lines are specified by type of treatment (e.g. first line chemotherapy, first line ET).

<u>Number of new patients eligible for later line therapy with CDK4/6</u>

Patients that are eligible for treatment with CDK4/6 in the second line of ET treatment include 1) patients treated with first line ET and 2) patients treated with first line chemotherapy receive subsequent treatment with ET.

- Of group 1 (patients treated with first line ET) it is expected that 58% will receive second line ET, 34% first line chemotherapy and 8% no treatment. These estimates are based on the pivotal RCT (PALOMA 2) of palbociclib + letrozole by Rugo et.[11] In this study, 58% of patients treated in the placebo arm (ET only) received ET as subsequent treatment and 34% chemotherapy. Based on expert opinion, we assumed that approximately 90% of 58% (n=760) is eligible for treatment with CDK4/6 + ET as the 2nd line ET treatment.
- A proportion of patients of group 2 (treated with first line ET and thereafter with chemotherapy) is also eligible for another line of ET. Based on the study of Fietz et all we estimated that this would be approximately 35%.[15] In figure 6a of this study it is reports that 32% of patients receives chemotherapy as third line therapy (third line is irrespective of type of therapy) without describing what their previous therapy was. The figure also reports that approximately 33% of patients died or were lost to follow-up after their 2nd line of treatment. The remaining 35% is eligible for 2nd line ET + CDK4/6 and we assumed that 70% of these patients would receive ET+CDK4/6 (n=121).

Additionally, patients treated with first line chemotherapy followed by first line ET are also eligible for 2nd line CDK4/6+ET. We estimated that this are 15 patients by applying the same proportions as applied to group 2 (described above).

In total 760 + 121 + 15 = 895 patients would be eligible for treatment with CDK4/6 in the second line implying 75 patients per month.

CDK4/6 inhibitors for breast cancer – Prevalent cases

© 2023 Luyendijk M et al. *JAMA Network Open.*

Prevalent cases per month are the number of new patients added to the patients that did not progress from the previous month. To calculate this we used the probabilities to progress obtained from PALOMA 2 (Palbociclib + AI) and 3 (Palbociclib + fulvestrant), for first and second line treatment respectively.[6,7] This approach was used to calculate the treated patients during the early access phase. In this phase we received information of the incident cases from the manufacturer (see eAppendix 2). This approach was also used to calculate the prevalent estimated eligible patients. For this purpose the incident cases as calculated above were used.

Original Kaplan-Meier curves and extrapolations of the PALOMA 2 – first line CDK4/6

eFigures 3 and 4 shows the original Kaplan-Meier curves reported in the publication of the PALOMA 2 and the simulated Kaplan-Meier curves and their extrapolations.[6] The Kaplan-Meier curve of the Palbociclib+letrozole arm of this study was simulated using the WebPlotDigitizer software. After having simulated the curves we extrapolate the survival curves by fitting different parametric functions (see eFigure 4). The parametric functions were compared to the Kaplan-Meier curve and based on visual inspection and the Akaike Information Criteria and expert opinion we chose the best fitting and most plausible function. For the PALOMA 2, this was the Weibull distribution. Based on the simulated Kaplan-Meier curve we calculated the probabilities to progress before 29 months and based on the Weilbull distribution we calculated the probabilities to progress beyond 29 months(up until 61 months i.e. the end of December 2021).

© 2023 Luyendijk M et al. *JAMA Network Open.*

eFigure 3. Original Kaplan-Meier Curves of the PALOMA-2

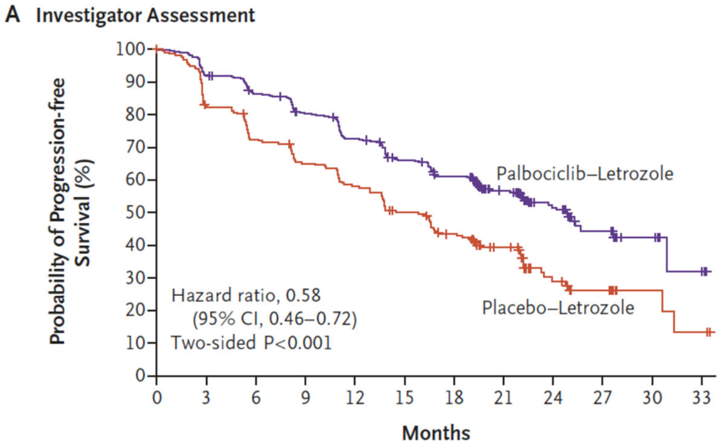

No. at Risk												
Palbociclib–Letrozole	444	395	360	328	295	263	238	154	69	29	10	2
Placebo–Letrozole	222	171	148	131	116	98	81	54	22	12	4	2

© 2023 Luyendijk M et al. *JAMA Network Open.*

eFigure 4. Simulated Kaplan-Meier Curves of the PALOMA-2 and the Extrapolated Curves Based on Different Distributions

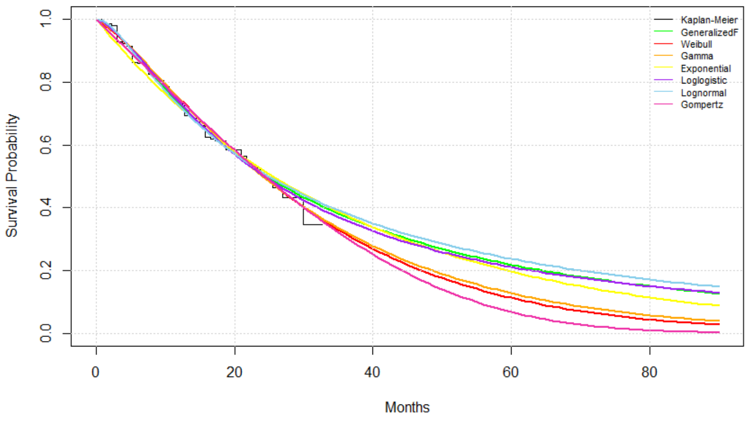

<u>Original Kaplan-Meier curves and extrapolations of the PALOMA 3 – second line CDK4/6</u>

eFigures 5 and 6 shows the original Kaplan-Meier curves reported in the publication of the PALOMA 3 and the simulated Kaplan-Meier curves and their extrapolations.[7] We used the same approach as for the PALOMA 2 (described above). We chose the Gompertz distribution as the best fitting function. Based on the simulated Kaplan-Meier curve we calculated the probabilities to progress up until 11 months and based on the Gompertz distribution we calculated the progression probabilities beyond 11 months.

© 2023 Luyendijk M et al. *JAMA Network Open.*

eFigure 5. Original Kaplan-Meier curves of the PALOMA-3

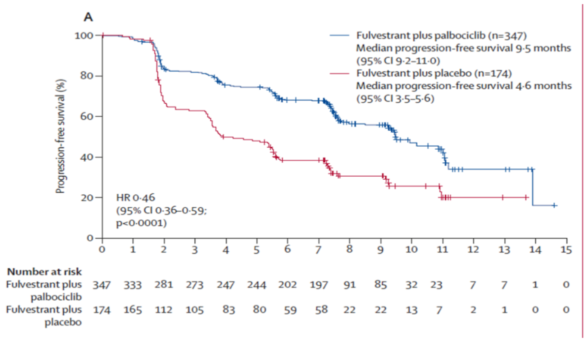

© 2023 Luyendijk M et al. *JAMA Network Open.*

eFigure 6. Simulated Kaplan-Meier Curves of the PALOMA-3 and the Extrapolated Curves Based on Different Distributions

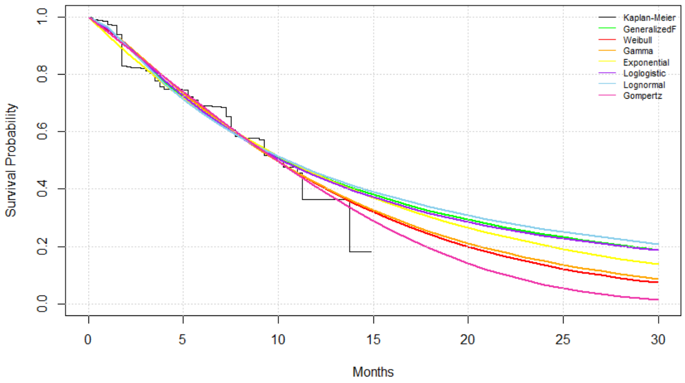

Uncertainty of the estimated eligible patients

To represent the impact of uncertainty on the estimates of number of patients, different scenario analyses were performed. We computed an upper and lower bound by changing the estimated new patients eligible per month (incident cases) as these estimates were considered the most uncertain parameters of the model. We estimated that each month 198 new patients with HR+/HER2- metastatic breast cancer would be diagnosed. For the base case we assumed that of those, 57% would be eligible for CDK4/6 inhibitors of whom 19% for first and 38% for second line setting. For the lower, upper bound we changed the percentages eligible for treatment.

© 2023 Luyendijk M et al. *JAMA Network Open.*

eReferences

1. Netherlands Association of Medical Oncologists. Are the assessment criteria still up-to-date? (In Dutch: Zijn de PASKWIL-criteria anno 2007 nog up-to-date?). https://www.nvmo.org/bom/zijn-de-paskwil-criteria-anno-2007-nog-up-to-date/?meta. Published September 9, 2007. Accessed February 2, 2022
2. Enzing JJ, Knies S, Boer B, Brouwer WB. Broadening the application of health technology assessment in the Netherlands: a worthwhile destination but not an easy ride? Health Econ Policy Law. 2021;16(4): 440-56.
3. National Health Care Institute. Assessment procedure for specialist medicines [in Dutch: Beoordelingsprocedure specialistische geneesmiddelen]. https://www.zorginstituutnederland.nl/over-ons/werkwijzen-en-procedures/adviseren-over-en-verduidelijken-van-het-basispakket-aan-zorg/beoordeling-van-geneesmiddelen/beoordeling-dure-specialistische-geneesmiddelen. Published May 11, 2020. Accessed August 22, 2022.
4. Employee ZIN. Personal communicatie. 2022.
5. Ministry of Health the Netherlands. Description of processes add-on drugs and clotting factors [In Dutch: Beschrijving processen add-on geneesmiddelen en stollingsfactoren]. https://www.farmatec.nl/prijsvorming/add-on-geneesmiddelen-sluismiddelen/beschrijving-processen-add-on-geneesmiddelen-en-stollingsfactoren. Accessed February 2, 2021.
6. Finn RS, Martin M, Rugo HS, et al. Palbociclib and letrozole in advanced breast cancer. NEJM 2016; 375(20):1925-36.
7. Cristofanilli M, Turner NC, Bondarenko I, et al. Fulvestrant plus palbociclib versus fulvestrant plus placebo for treatment of hormone-receptor-positive, HER2-negative metastatic breast cancer that progressed on previous endocrine therapy (PALOMA-3): final analysis of the multicentre, double-blind, phase 3 randomised controlled trial. Lancet Oncol 2016; 17(4):425-39.
8. Hoyle MW, Henley W. Improved curve fits to summary survival data: application to economic evaluation of health technologies. BMC medical research methodology 2011;11(1):1-14.
9. Latimer NR. Survival analysis for economic evaluations alongside clinical trials—extrapolation with patient-level data: inconsistencies, limitations, and a practical guide. Med Dec Making 2013; 33(6): 743-54.
10. Medical Oncology. Positioning: application of palbociclib in breast cancer [in Dutch: Plaatsbepaling NABON en NVMO: toepassing van palbociclib bij mammacarcinoom].

© 2023 Luyendijk M et al. *JAMA Network Open.*

https://medischeoncologie.nl/artikelen/2017/februari/editie-1/plaatsbepaling-nabon-en-nvmo-toepassing-van-palbociclib-bij-mammacarcinoom. Published February 13, 2017. Accessed February 2, 2022.

11. Rugo H, Finn R, Diéras V, et al. Palbociclib plus letrozole as first-line therapy in estrogen receptor-positive/human epidermal growth factor receptor 2-negative advanced breast cancer with extended follow-up. Breast Cancer Res and Treat 2019;174(3): 719-29.

12. Lobbezoo D, Van Kampen R, Voogd A, et al. In real life, one-quarter of patients with hormone receptor-positive metastatic breast cancer receive chemotherapy as initial palliative therapy: a study of the Southeast Netherlands Breast Cancer Consortium. Ann Oncol 2016;27(2):256-62.

13. Netherlands Cancer Registry.

14. ZIN. Pakketadvies sluisgeneesmiddel palbociclib (Ibrance®) voor patiënten met lokaal gevorderde of gemetastaseerde borstkanker. 2017. https://www.zorginstituutnederland.nl/publicaties/adviezen/2017/04/11/pakketadvies-palbociclib#:~:text=Zorginstituut%20Nederland%20adviseert%20de%20minister,impact%20op%20het%20zorgbudget%20reduceert. Accessed February 2, 2022. .

15. Fietz T, Tesch H, Rauh J, et al. Palliative systemic therapy and overall survival of 1,395 patients with advanced breast cancer–results from the prospective German TMK cohort study. The Breast 2017;34:122-30.

© 2023 Luyendijk M et al. *JAMA Network Open.*
