# Supplementary material for: Regulatory Approval, Reimbursement, and Clinical Use of Cyclin-Dependent Kinase 4/6 Inhibitors in Metastatic Breast Cancer in the Netherlands
Source: JAMA Netw Open. 2023 Feb 16;6(2):e2256170. doi: 10.1001/jamanetworkopen.2022.56170 (PMC9936344; doi:10.1001/jamanetworkopen.2022.56170)
Supplement: Supplement 2. — Data Sharing Statement [file jamanetwopen-e2256170-s002.pdf]

Data Sharing Statement

Luyendijk. Regulatory Approval, Reimbursement, and Clinical Use of Cyclin-Dependent Kinase 4/6 Inhibitors in Metastatic Breast Cancer in the Netherlands. *JAMA Netw Open*. Published February 16, 2023. doi:10.1001/jamanetworkopen.2022.56170

Data
Data available: No

Additional Information
Explanation for why data not available: The data analyzed for this study are available from the Dutch Hospital Data (L20.037 & L.22.035)
